# Laparoscopic Versus Open Surgical Management of Hydrocele of the Canal of Nuck: A Retrospective Analysis of 20 Cases

**DOI:** 10.7759/cureus.56584

**Published:** 2024-03-20

**Authors:** Rajalakshmi Venkateswaran, Kashif Ansari, Supriya Bhondve, Ajay Bhandarwar, Harshal D Padekar, Snehal Dandge, Amit V Dashputra

**Affiliations:** 1 General Surgery, Grant Government Medical College and Sir JJ (Jamshedjee Jeejeebhoy) Group of Hospitals, Mumbai, IND

**Keywords:** trans-abdominal pre-peritoneal approach, open anterior approach, magnetic resonance imaging, processus vaginalis, hydrocoele of canal of nuck

## Abstract

Introduction: Hydrocele of the canal of Nuck is a condition that arises due to incomplete obliteration of the processus vaginalis or an abnormal outpouching from the round ligament during fetal development. It usually presents as a painless, rarely painful, groin swelling. The definitive diagnosis for this condition is magnetic resonance imaging. Various management options have been proposed for this condition, including open surgery, transabdominal preperitoneal approach, totally extraperitoneal approach, and a combination of laparoscopic and open surgery. The present study highlights the benefits of the transabdominal preperitoneal approach when compared with the open anterior approach and addresses the intraoperative challenges faced during laparoscopic surgery.

Materials and methods: The study is a retrospective study inclusive of 20 patients who underwent surgery for the hydrocele of the canal of Nuck from June 2019 to December 2023. Case records of patients were studied for information such as demographic features, type of pathology, the surgery performed, intraoperative challenges encountered, operative time, duration of hospital stay, scores from the visual analog scale pain assessment chart at various intervals, and time taken to return to work. The variables were documented and statistically analyzed.

Results: The average age group of the study population was 27.8 ± 8.34 years. Of the 20 patients, 10 had undergone a transabdominal preperitoneal approach (Group A), and 10 had undergone an open anterior approach (Group B). Eleven out of 20 patients had an associated inguinal hernia, of which three were identified preoperatively and eight were identified incidentally during surgery. The mean operative time of Group A cases was 97.95 ± 7.54 minutes, while it was 66.3 ± 6.20 minutes for Group B cases. The Mann-Whitney U test showed a statistically significantly lesser operative time for Group B than for Group A (p-value < 0.001). The duration of hospital stays was comparable for the two groups with no significant difference (two days versus 3.8 ± 3.08 days, respectively). When the difference in the means of time taken to return to normal work was compared using the Mann-Whitney U test between Group A and B (6.1 ± 0.87 days and 11.2 ± 1.81 days, respectively), a statistically significant early return to normal work in the former group (p-value = 0.001) was revealed. Similarly, the Mann-Whitney U test when used to compare the median postoperative pain score of both groups at 12-24 hours, 48-72 hours, seven days, and three months showed a significantly lesser pain score among patients of Group A at all intervals (p-value < 0.001, p-value = 0.005, p-value = 0.005, p-value < 0.001, respectively). The incidence of intraoperative challenges, sero-hematoma, and surgical site infection were insignificant in comparison.

Conclusion: The transabdominal preperitoneal approach for the hydrocele of the canal of Nuck is ideal as it offers excellent intraoperative delineation of pathology and postoperative outcomes. Prophylactic placement of a mesh in all cases can help prevent a future occurrence of inguinal hernia in these cases.

## Introduction

Canal of Nuck pathologies are rare clinical conditions seen in females, arising due to the failure of its obliteration. The canal of Nuck is formed by a fold of parietal peritoneum that is attached to the round ligament and is homologous to the processus vaginalis in males [1]. In cases where this canal remains patent in adulthood, fluid, bowel, or even the ovaries can form its content, presenting as a painless or painful, cystic to firm swelling in the groin.

Hydrocele of the canal of Nuck (HCN) is the term used for the condition wherein fluid occupies a patent canal [2]. There are two theories behind the pathogenesis of this condition. According to the first theory by Dutch anatomist Anton Nuck, HCN arises as a result of the failure of obliteration and abnormal outpouching of the canal of Nuck [3]. The second theory attributes this to be a remnant cyst arising from the round ligament during its developmental phase [4]. The differential diagnosis of this condition includes a Bartholin’s cyst, hematoma, inguinal or femoral hernia, endometriosis of the canal of Nuck, leiomyoma, metastasis, etc. [3,5].

A magnetic resonance imaging (MRI) scan is ideal for diagnosing this condition as it helps to clearly define the extent and composition of soft tissue lesions [6]. The management options discussed in the literature for HCN include complete excision through an open anterior approach and laparoscopic surgery. While both approaches have their own advantages and complications, laparoscopic management has the added benefits of less pain, small scars, and better delineation of anatomy. The present study compares outcomes of the transabdominal preperitoneal (TAPP) approach versus open surgery in cases of HCN in terms of the below-mentioned operative and patient parameters.

## Materials and methods

Case records of all female patients who underwent surgery for HCN in a tertiary care hospital in Mumbai during the period June 2019 to December 2023 were retrospectively analyzed in this study. The study group excluded pregnant patients and those who had previously undergone abdominal surgeries. A total of 20 patients formed the study group. Patients who underwent the TAPP approach were categorized as Group A and those who underwent open surgery were categorized as Group B. All patients were operated by surgeons with a similar experience. On analysis, 10 patients who underwent open surgery were patients with respiratory illnesses and hence were deferred general anesthesia. All patients had an MRI done before surgery, which was suggestive of a probable diagnosis of HCN. Besides demographic data, the type of HCN in each case, the procedure performed, intraoperative challenges, operative time (skin incision to skin closure), duration of hospital stay, postoperative pain score, and time taken to return to work were documented and analyzed from the records of these patients. Intraoperative challenges faced were noted such as injury to the inferior epigastric artery (IEA), difficulty in dissecting the distal end of the cyst, and the need for conversion from laparoscopic to open surgery. The post-surgery pain scores marked in the visual analog scale (VAS) pain assessment chart present in each patient record (at 12-24 hours, 48-72 hours, one week, and three months) were documented and analyzed using various tests.

Histopathological analysis of all cysts revealed mesothelial cysts with no malignant cells or endometriosis. All patients had an uneventful postoperative period.

Surgical procedures

TAPP

**Figure 1:**
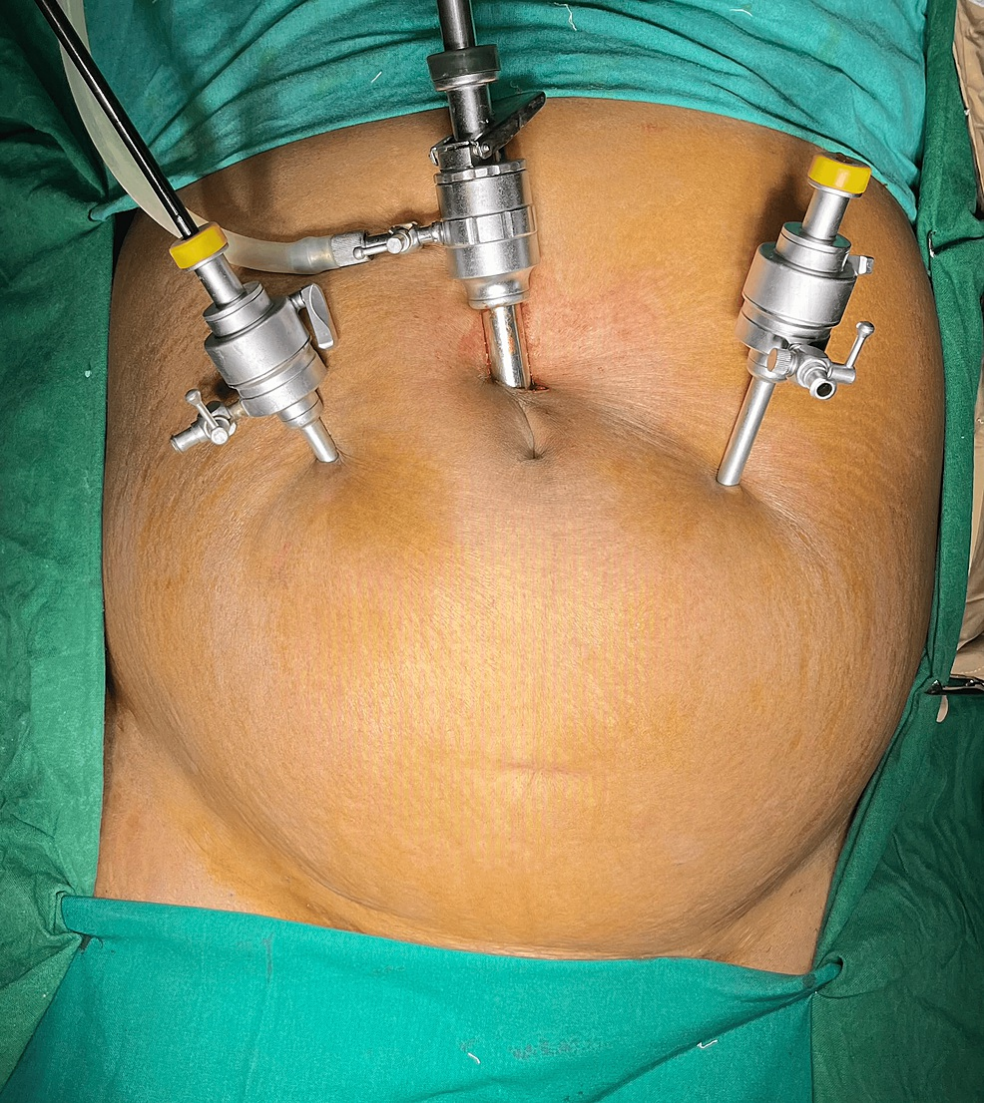
Port placement for TAPP approach. A 10-mm camera port was placed 1.5 cm above the umbilicus, and two 5-mm working ports were placed as shown in the clinical image, 5 cm lateral to the umbilicus TAPP: Transabdominal preperitoneal.

The procedure was done under general anesthesia. The patient was placed in a supine position with the head end lowered. The surgeon operated from the head end of the patient. Ports were inserted according to Figure 1.

**Figure 2:**
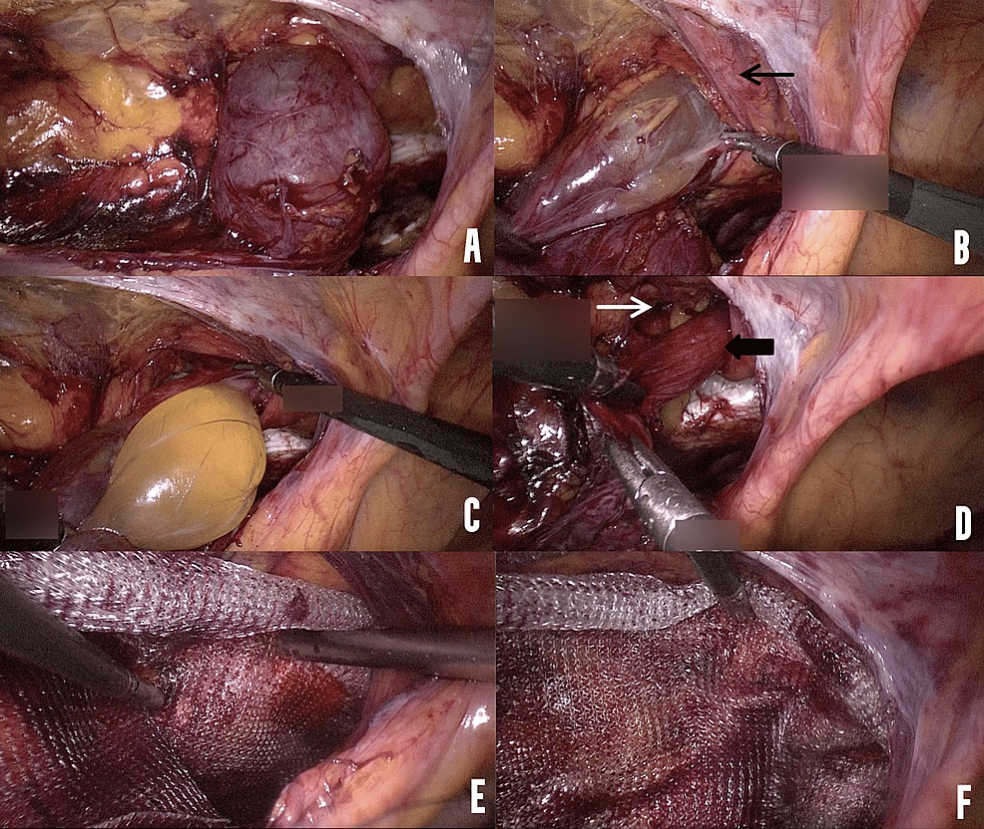
Steps of TAPP approach to HCN. (A) Cyst seen entering the deep ring. (B) Lateral traction was applied, and the cyst was separated from the artery and attached to the fascia transversalis. The black arrow indicates the IEA. (C) The distal most part of the cyst was dissected. (D) An indirect hernial sac is seen entering the internal ring. The black arrow shows the hernial sac, and the white arrow shows the widened internal ring. (E) Polypropylene mesh was fixed medially to Cooper’s ligament. (F) Polypropylene mesh covered the defect in the deep ring. TAPP: Transabdominal preperitoneal; IEA: Inferior epigastric artery; HCN: Hydrocele of the canal of Nuck.

A peritoneum flap was created, and HCN was identified (Figure 2, Panel A). The cyst was then retracted laterally, and adhesions between the cyst and the region of the IEA were separated by pulling the fibers medially (Figure 2, Panel B). No energy source was utilized to separate the HCN from the region of the IEA unless there was bleeding in which case a bipolar energy device was utilized. The distalmost end of the cyst which was within the inguinal canal was also separated from its attachments to the abdominal wall by blunt dissection and sufficient proximal traction to the cyst wall (Figure 2, Panel C). The cyst was excised in toto. The hernial sac and widened deep ring were identified (Figure 2, Panel D), and the sac was separated from the round ligament. A polypropylene mesh was then placed in the preperitoneal plane (Figure 2, Panels E and F) and fixed to Cooper’s ligament medially. The peritoneal flap was closed with an absorbable suture. The cyst was removed in toto.

Open Anterior Approach (OAA)

All patients were administered spinal anesthesia. An incision was taken parallel to the inguinal ligament, about 1.5 cm above it. An incision was deepened in layers, and external oblique aponeurosis was opened. The cyst was identified; its extent was defined and then separated from the round ligament. A hernia sac, if present, was separated from the round ligament, transfixed, and repositioned into the peritoneal cavity. The internal ring defect was repaired, and a polypropylene mesh was placed on the posterior wall of the inguinal canal. The mesh was secured medially to the rectus muscle and laterally to the upturned part of the inguinal ligament. The abdominal wall was closed in layers.

Statistical analysis

The data was collected and entered into Microsoft Excel (Microsoft Corp., Redmond, Washington) and analyzed using IBM SPSS software, version 21 (IBM Corp., Armonk, NY). The data was presented as percentages and categories and then presented as tables. The Mann-Whitney U test was used for comparison of variables and test of significance. A p-value of less than 0.05 was considered statistically significant.

## Results

A total of 20 female patients were included in this study. The average age group of the study population was 27.8 ± 8.34 years. Of them, 10 patients had undergone the TAPP approach, and 10 patients had undergone OAA, who were then categorized into Group A and Group B, respectively.

In Group A, seven patients had type 1 HCN, and three had type 3 HCN, whereas in Group B, eight patients presented with type 1 HCN, and two patients with type 3 HCN. Overall, 11 out of the 20 cases had an associated indirect inguinal hernia, of which three were diagnosed preoperatively and eight were identified incidentally during surgery. Seven patients of Group A and four patients of Group B had an associated inguinal hernia.

**Figure 3:**
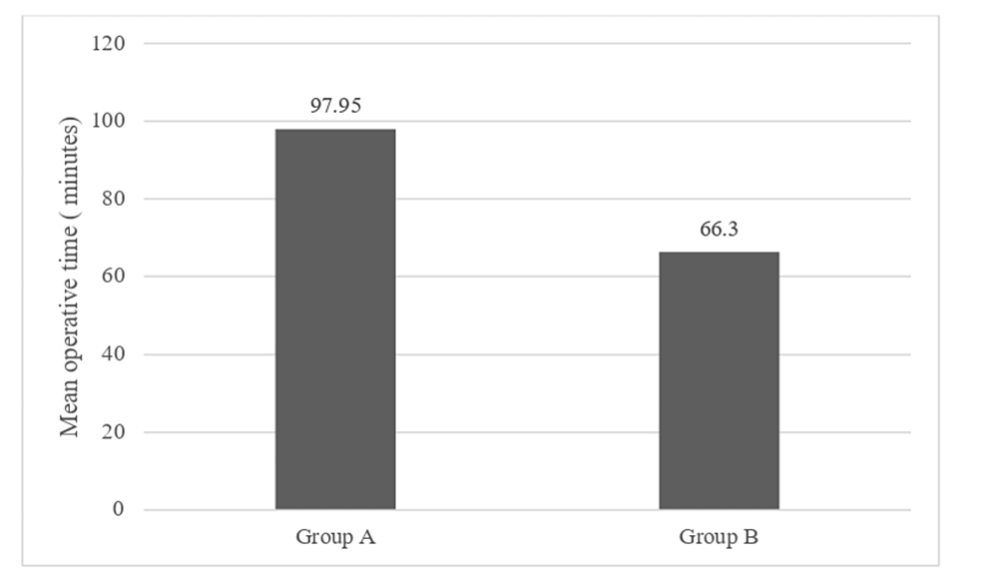
Mean operative time among patients of Group A and Group B The mean operative time was lesser for Group B patients as compared to patients of Group A when compared using the Mann-Whitney U test (Z = -3.781, p-value < 0.001).

The mean operative time of Group A cases was 97.95 ± 7.54 minutes, while it was 66.3 ± 6.20 minutes for Group B patients (Figure 3). The data, when analyzed using the Mann-Whitney U test, showed a statistically significantly lesser operative time for Group B when compared to Group A (Z = -3.781, p-value < 0.001). The average duration taken to operate cases of type 3 HCN in Group A and Group B was 108 and 76.75 minutes, respectively.

**Figure 4:**
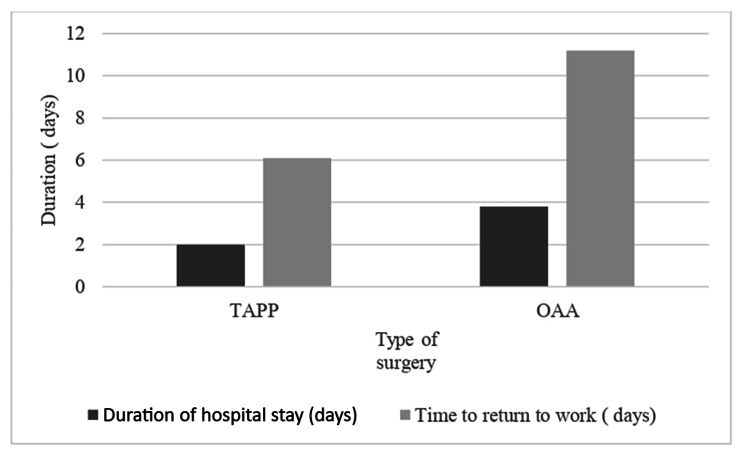
Duration of hospital stay and time taken to return to work The Mann-Whitney U test revealed that the time taken to return to work was significantly less for patients in Group A when compared to Group B (Z = -3.841, p-value < 0.001). TAPP: Transabdominal preperitoneal; OAA: Open anterior approach.

The duration of hospital stays and "time taken to return to work" for both groups were described in Figure 4. Patients of Group A had a shorter duration of hospital stay (two days versus 3.8 ± 3.08 days) and "time taken to return to work" (6.1 ± 0.875 days versus 11.2 ± 1.81 days) as compared to Group B. However, the Mann-Whitney U test showed a statistically significant difference only in the means of the two groups with respect to "time taken to return to work" (Z = -3.841, p-value < 0.001). The Mann-Whitney U test revealed a p-value of 0.068 when the means of the duration of hospital stay were compared.

The postoperative pain score and complications are listed in Table 1. The average pain scores at all intervals were lesser for patients of Group A as compared to Group B (Table 1). The Mann-Whitney U test, when applied to analyze the median values of the post-surgery pain scores between Group A and Group B, showed a statistically significant lesser pain score value in Group A at 12-24 hours, 48-72 hours, seven days, and three months (p-value < 0.001, p-value = 0.006, p-value = 0.005 and p-value < 0.001, respectively). The incidence of SSI or sero-hematoma when compared was not statistically significant.

**Table 1:** Assessment of postoperative parameters The Mann-Whitney U test showed a significantly lesser pain score in Group A patients as compared to Group B patients with a p-value of <0.05 in all compared intervals. TAPP: Transabdominal preperitoneal; OAA: Open anterior approach; IEA: Inferior epigastric artery; NA: Not applicable; SSI: Surgical site infection. *Standard deviation.

Parameters		TAPP (10 patients)	OAA (10 patients)	Statistics value	p-value
Intraoperative challenges	Injury to IEA	1	0	-	-
	Difficulty in tracing the distal end of the sac	2	NA	-	-
	Conversion to open	0	NA	-	-
Pain score (visual analog scale) (mean ± SD*)	12-24 hours	4.5 ± 0.527	6.3 ± 0.483	Z = -3.390	<0.001
	48-72 hours	3.3 ± 0.483	4.1 ± 0.567	Z = -2.768	0.006
	7 days	1.7 ± 0.483	2.5 ± 0.527	Z = -2.805	0.005
	3 months	0.3 ± 0.483	1.9 ± 0.567	Z = -3.743	<0.001
Incidence of sero-hematoma		1	3	Z = -1.090	0.276
Incidence of SSI		1	2	Z = -0.610	0.54

## Discussion

The hydrocoele of the canal of Nuck, also known as the female hydrocoele [7], is a rare clinical condition that is more often seen in younger females and corresponds to the spermatic cord hydrocoele found in males [3]. Based on anatomical variations, it is divided into three types: type 1, an encysted hydrocoele due to partial obliteration of the proximal portion of the canal of Nuck; type 2, the communicating type where the canal is in continuity with the peritoneum; and type 3, the combined type where there is a distal encysted portion as well as a proximal intra-abdominal portion, which is hourglass shaped due to a constriction at the internal ring [8].

Wang et al. described certain challenges faced during laparoscopic management of HCN in their publication such as interference of IEA and inability to access the distalmost end of the cyst [9]. We recommend that the separation of the cyst from the vicinity of the IEA can be done by lateral traction to the cyst and countertraction on the adhesions with minimal to nil usage of an energy device. However, if bleeding is encountered, it should be tackled with a bipolar energy device, instead of a monopolar energy source.

A case report published in 2019 by Chihara et al. described the "open posterior wall" technique to tackle a type 1 or 3 HCN [10]. This technique is undoubtedly beneficial, but it comes with the cost of creating an iatrogenic defect in an already weakened region of the abdominal wall. Hence, based on our experience in handling these cases, firm and adequate traction over the cyst or the round ligament is sufficient to visualize the portion of the cyst within the inguinal canal as most cases have a widened internal ring. This technique was applied successfully in all cases of our study whereby all cysts were excised en bloc without rupture.

The widening of the internal ring due to the cyst results in a high percentage of associated inguinal hernias, at 30%-50% [4]. Additionally, in the present study, inguinal hernias were detected during surgery in 16% of patients. Hence, reinforcing the posterior wall with the placement of a mesh should be done in all cases, irrespective of the presence or absence of a hernia. This can prevent a post-surgical incidence of hernia.

Comparative studies on outcomes of laparoscopic versus open surgical management of HCN are lacking in literature due to it being a rare clinical entity; hence, studies involving TAPP procedures for inguinal hernias were analyzed for the present study. Notably, TAPP has several advantages over open surgery, including better delineation of pathology [11], less pain [12], etc. Scheuermann et al. conducted a meta-analysis in 2017, which showed that the incidence of acute and chronic pain was significantly lesser in the patients who underwent TAPP (p-value = 0.005 and p-value = 0.006, respectively) as compared to the group who underwent Lichtenstein’s repair for inguinal hernia [13]. Another study conducted by Lin et al. in August 2023 comprised 159 female patients who underwent TAPP [14]. The results of the study showed that the pain score on POD 1 calculated by the VAS was 2.5 ± 0.7, and the mean length of hospital stay was 1.1 ± 0.2 days [14]. These studies along with the present study further augment the advantages of TAPP.

Secondly, the time taken to return to work is lesser in patients who undergo minimal access surgery as compared to open surgery [13,15,16]. A randomized control study conducted in 2020 compared outcomes among 60 patients who underwent bilateral TAPP and 60 who underwent Lichtenstein’s tension-free repair, which showed the time taken to return to normal work being 5.87 ± 0.97 and 12.10 ± 1.02 days, respectively, for both groups [15]. The results were comparable to the present study and can be explained as a result of decreased pain, smaller scar, and early recovery after surgery in any case of minimally invasive surgery.

Shortcomings of laparoscopic repair of HCN include a prolonged operative time, which is usually seen in type 1 and type 3 HCN. The portion of the HCN distal to the superficial ring is the major concern for most surgeons doing a TAPP. Rupture of the cyst can occur in such cases, which then creates a dilemma regarding mesh placement. Incomplete excision of the cyst can result in missing out on occult pathologies like endometriosis and malignancies. Hence, meticulous dissection and minimal use of energy sources can help deliver the cyst en bloc.

In our opinion, the TAPP approach for HCN should be considered in all patients despite its limitations as the post-procedure outcomes are better than an open surgical approach. Moreover, solutions to tackle intraoperative challenges, if applied, can help prevent complications and restrict the operative time.

To our knowledge, the present study is one of the very few studies that compares outcomes between open versus TAPP approach for HCN. Limitations of this study are a small sample size and a unicentric study. Larger multicentric studies are required to validate the results better.

## Conclusions

HCN, being a rare entity, has multiple approaches under trial for its management. TAPP helps visualization of the extent and anatomy of the cyst while ruling out any other intra-abdominal pathologies. Intraoperative challenges can be tackled with meticulous dissection, employing the correct technique of traction-countertraction, and avoiding inadvertent use of energy sources while dissecting the HCN away from the IEA.

TAPP, like any other minimally invasive surgery, is significantly superior to OAA in terms of lesser pain, smaller scar, and early return to work. Hence, we recommend proceeding with TAPP for all cases of HCN. Placement of a mesh in the preperitoneal plane in all cases should be done to prevent the development of an inguinal hernia.

## References

[1] Kim KS, Choi JH, Kim HM, Kim KP, Kwon YJ, Hwang JH, Lee SY (2016). Hydrocele of the canal of Nuck in a female adult. Arch Plast Surg.

[2] Keeratibharat N, Chansangrat J (2022). Hydrocele of the canal of Nuck: a review. Cureus.

[3] Kohlhauser M, Pirsch JV, Maier T, Viertler C, Fegerl R (2022). The cyst of the canal of Nuck: anatomy, diagnostic and treatment of a very rare diagnosis-a case report of an adult woman and narrative review of the literature. Medicina (Kaunas).

[4] Manatakis DK, Stamos N, Agalianos C, Vamvakas P, Kordelas A, Davides D (2013). Mesothelial cyst of the round ligament misdiagnosed as irreducible inguinal hernia. Case Rep Surg.

[5] Sarkar S, Panja S, Kumar S (2016). Hydrocele of the canal of Nuck (female hydrocele): a rare differential for Inguino-labial swelling. J Clin Diagn Res.

[6] Fikatas P, Megas IF, Mantouvalou K (2020). Hydroceles of the canal of Nuck in adults-diagnostic, treatment and results of a rare condition in females. J Clin Med.

[7] Kono R, Terasaki H, Murakami N, Tanaka M, Takeda J, Abe T (2015). Hydrocele of the canal of Nuck: a case report with magnetic resonance hydrography findings. Surg Case Rep.

[8] Gkioulos F, Theodoridou S, Abay B, Engledow AH (2023). A case report of female hydrocele of the canal of Nuck (type I): a diagnostic challenge and surgical solution. Cureus.

[9] Wang L, Maejima T, Fukahori S, Shun K, Yoshikawa D, Kono T (2021). Laparoscopic surgical treatment for hydrocele of canal of Nuck: A case report and literature review. Surg Case Rep.

[10] Chihara N, Taniai N, Suzuki H, Nakata R, Shioda M, Yoshida H (2020). Use of a novel open posterior wall technique for laparoscopic excision of hydrocele of the canal of Nuck in an adult female: case report. J Nippon Med Sch.

[11] Shahid F, El Ansari W, Ben-Gashir M, Abdelaal A (2020). Laparoscopic hydrocelectomy of the canal of Nuck in adult female: case report and literature review. Int J Surg Case Rep.

[12] Haladu N, Alabi A, Brazzelli M, Imamura M, Ahmed I, Ramsay G, Scott NW (2022). Open versus laparoscopic repair of inguinal hernia: an overview of systematic reviews of randomised controlled trials. Surg Endosc.

[13] Scheuermann U, Niebisch S, Lyros O, Jansen-Winkeln B, Gockel I (2017). Transabdominal preperitoneal (TAPP) versus Lichtenstein operation for primary inguinal hernia repair - A systematic review and meta-analysis of randomized controlled trials. BMC Surg.

[14] Lin R, Lin X, Yang Y (2023). Laparoscopic transabdominal preperitoneal repair for female patients with groin hernias. BMC Womens Health.

[15] Nair CC, Karthikeyan EM, Dhyaneshwaran KV, Selvaraj N (2021). Surgical sequale of transabdominal preperitoneal approach versus Lichtenstein open repair in a rural setting. International Surgery Journal.

[16] Elmessiry MM, Gebaly AA (2020). Laparoscopic versus open mesh repair of bilateral primary inguinal hernia: a three-armed Randomized controlled trial. Ann Med Surg (Lond).

